# Clinical and Behavioral Correlates of Blood Acylcarnitine Profiles in Children with Autism Spectrum Disorder: A Cross-Sectional Analysis

**DOI:** 10.3390/children12070848

**Published:** 2025-06-27

**Authors:** Adriana Prato, Martina Randazzo, Maria Anna Messina, Giovanni Puglisi, Laura Rosy Aleo, Fiorella Ciantia, Lara Cirnigliaro, Renata Rizzo, Rita Barone

**Affiliations:** 1 Child and Adolescent Neurology and Psychiatric Section, Department of Clinical and Experimental Medicine, Catania University, 95123 Catania, Italy; adrianaprato01@gmail.com (A.P.); randazzo97@gmail.com (M.R.); giovanni97mail@gmail.com (G.P.); lauraaleo9898@gmail.com (L.R.A.); fiorella.ciantia@gmail.com (F.C.); lara.cirnigliaro@phd.unict.it (L.C.); rerizzo@unict.it (R.R.); 2 Expanded Newborn Screening Laboratory, A.O.U Policlinico “G. Rodolico—San Marco”, 95123 Catania, Italy; mmessina@unict.it; 3 Research Unit of Rare Diseases and Neurodevelopmental Disorders, Oasi Research Institute-IRCCS, 94018 Troina, Italy

**Keywords:** autism spectrum disorder, ADOS, dried blood spots, ESI-MS/MS, fatty acid oxidation, metabolism, acylcarnitines, medium-chain triglycerides

## Abstract

**Background/Objectives**: Autism Spectrum Disorder (ASD) etiology is complex, involving genetics and environmental factors, and associated with impaired energy metabolism. Mitochondrial fatty acid oxidation (mFAO) is instrumental to energy production through the oxidation of acylcarnitines (ACs). We performed a comprehensive investigation of blood AC profiles in a pediatric ASD cohort, aiming to define ASD subgroups based on AC profiles and link these profiles to key clinical features and comorbidities using a phenotype-first approach. **Methods**: Blood levels of 31 ACs (μmol/L) collected from 102 ASD patients and 117 healthy controls (HCs) were evaluated via tandem mass spectrometry. The percentile distribution of blood AC levels in HC samples was computed to define the normal reference range (RR) and identify values corresponding to the 10th and 90th percentiles. Cognitive levels, emotional–behavioral disturbances and the severity of ASD symptoms (Autism Diagnostic Observation Schedule-Calibrated Severity Score ADOS-CSS) were assessed. Clinical correlates of ASD groups based on AC profiles were evaluated. **Results**: Three ASD subgroups were identified based on the percentile distribution of AC levels: group A (ACs < 10th percentile), group B (ACs 10th–90th percentile) and group C (ACs > 90th percentile) (abnormal AC number ≥ 3). Out of the thirty-one analyzed ACs in DBSs, fifteen (48.4%) were significantly different when comparing ASD group A to ASD group C. There was a significant difference in the severity of autism symptoms (ADOS CSS) related to the repetitive and restricted behaviors domain (CSS RRB) among the different groups (χ^2^(2) = 6.26; *p* = 0.044). The post hoc Dunn’s test with Bonferroni correction showed that ADOS-CSS RRB was significantly higher in ASD group A compared to ASD group B (*p* = 0.013). AC C14 was more frequently decreased (<10th pc) in patients with more severe symptoms (*p* = 0.006); C10:1 tended to be more frequently increased (>90th pc) in patients with lower clinical severity (*p* = 0.052). **Conclusions**: This study highlights differences across blood AC levels in children with ASD and conveys novel information on clinical severity in ASD patients with abnormal blood AC profiles. Thus, examining metabolic profiles may provide helpful insights to understand the variability of ASD symptoms.

## 1. Introduction

Autism Spectrum Disorder (ASD) is a neurodevelopmental condition marked by challenges in social communication and interaction across various settings, along with repetitive and restricted behaviors, interests or activities [1]. The current prevalence of ASD is approximately 1 in 36 (2.8%) among 8-year-old children in the U.S. [2], and it is higher in males than in females (3.7% in boys and 0.9% in girls aged 8 years) [3]. The majority of children diagnosed with ASD present with at least one additional condition or symptom, such as Attention Deficit Hyperactivity Disorder (ADHD), intellectual disability (ID), epilepsy, sleep disorder and gastrointestinal disorder [4,5]. ASD etiology is complex, involving genetic and environmental contributors, and no biological markers are available for diagnosis and outcome predictions so far. Among different biological causes, energy metabolism disarrangement is indicated by the occurrence of various mitochondrial biomarkers in tissues and biological fluids from patients with ASD [6,7,8]. Specifically, increased lactate, pyruvate, alanine, alanine aminotransferase, alanine-to-lysine ratio and acylcarnitine (AC) levels have been detected, on average, in 30–50% of individuals with ASD [6,9]. ASD has been associated with abnormal citric acid (TCA) cycle metabolites, defective electron transport chain (ETC) complex activity in immune cells [10] and increased activity in the ETC complexes and mitochondrial respiration, enhancing sensitivity to physiological stressors and predisposing individuals to regression [11]. Consequently, individuals with ASD seem to exhibit distinct mitochondrial metabolic abnormalities. However, most patients do not fulfill the diagnostic criteria for primary mitochondrial disease, and genetic abnormalities are often absent, so it is reasonable to hypothesize that this may be related to a secondary mitochondrial dysfunction [7].

The mitochondrial fatty acid beta-oxidation (mFAO) pathway serves to progressively shorten long, medium and short fatty acids conjugated with carnitine (ACs) within the energy metabolism. Inborn errors of metabolism affecting carnitine and mFAO are characterized by energy deficiency leading to brain and multi-system pathology [12]. The occurrence of specific, abnormal blood AC profiles in primary genetic defects of mFAO indicates that blood AC levels reflect the physiological functioning of mFAO and energy metabolism. Among metabolic ASD biomarkers, abnormal AC profiles were reported in uncontrolled and controlled studies, indicating the disturbance of mFAO in ASD. Based on the repeated occurrence of abnormal values in at least three individual ACs, the prevalence of abnormal ACs in ASD patients from two different studies was 19% [13,14]. Two controlled studies (ASD children vs. healthy controls) found a unique AC profile in ASD with increased short and long AC elevations [12,14]. Furthermore, by comparing ASD children with and without ACs elevations, significant differences were found in several short- and medium-chain ACs and various long-chain ACs [7,15,16]. These findings suggest that nearly one-fifth of ASD patients exhibit distinctive changes in their AC profiles, which are not caused by primary defects in mFAO. Distinctive blood AC profile abnormalities might represent potential biomarkers for acquired mitochondrial disease in ASD and may also reflect underlying biochemical heterogeneity within the disorder.

Expanding on these metabolic insights, functional studies performed on fibroblasts derived from ASD patients revealed that those with elevated blood AC levels exhibited significantly reduced mFAO and ETC activity compared to patients with normal AC profiles. These results imply a direct impairment of mitochondrial energy metabolism in a subgroup of ASD patients, potentially contributing to the clinical manifestations of the disorder. Moreover, resveratrol (RSV), a natural polyphenol with antioxidant and mitochondrial-enhancing properties, was observed to significantly increase mFAO activity in fibroblasts from both groups, especially in those with more severe ASD symptoms [16]. In sum, previous studies have provided concrete evidence linking AC abnormalities and disturbed energy metabolism to ASD. Therefore, RSV or similar mitochondrial-targeted interventions could offer a promising avenue for modulating metabolic dysfunction in ASD, especially in individuals characterized by specific metabolic profiles.

In this regard, levels of free L-carnitine—a cofactor essential for shuttling very-long-chain and long-chain fatty acids across the inner mitochondrial membrane into the mitochondrial matrix, allowing their oxidation and energy production—and total L-carnitine concentrations were decreased in a large proportion of patients with ASD [17]. Moreover, elevated plasma concentrations of long-chain fatty acids have been observed in individuals with ASD [18]. Taken together, these studies not only reinforce the importance of metabolic alterations, particularly in AC levels and mitochondrial function, in ASD but also open new perspectives for personalized therapeutic strategies aimed at correcting mitochondrial deficits and improving clinical outcomes. Based on this evidence, the present study embarks on a comprehensive exploration of metabolic signatures, focusing on mFAO and the intricate relationship between AC profiles and ASD, in a cohort of pediatric participants. The primary goal was to identify distinct subgroups within the ASD cohort based on their AC profiles to enhance our comprehension of how established changes in AC blood profiles may relate to the clinical symptoms observed in ASD. Furthermore, using a phenotype-first approach, we aimed to evaluate whether pertinent clinical features and comorbidities of ASD subjects recruited for this study might be associated with distinct blood AC profiles.

## 2. Materials and Methods

### 2.1. Participants

**Figure 1:**
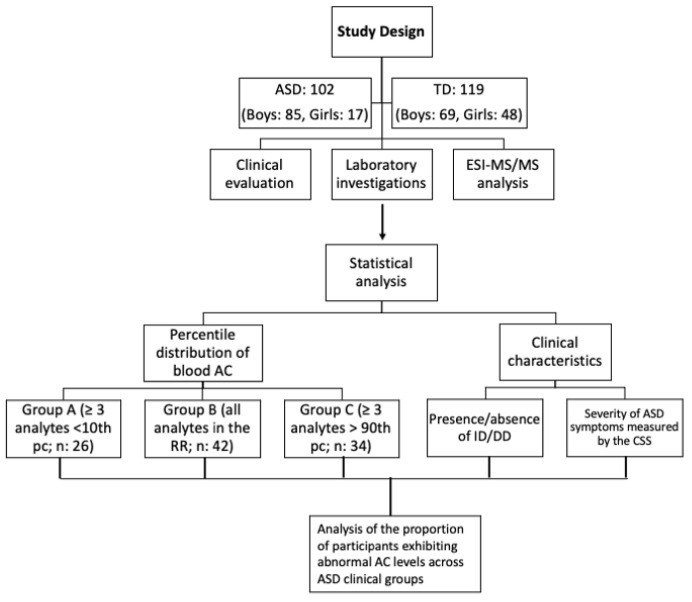
Diagram of the study design. ASD: Autism Spectrum Disorder; TD: Typically Developing; AC: Acylcarnitine; RR: Reference Range; ID: Intellectual Disability; DD: Developmental Delay; CSS: Calibrated Severity Score; ESI-MS/MS: electrospray ionization tandem mass spectrometry.

The present cross-sectional study (Figure 1) comprises 219 participants, 102 children (85 boys and 17 girls) diagnosed with ASD (mean age 6.2 ± 3.5 years) and 117 typically developing (TD) children (69 boys and 48 girls), with similar age distribution. All participants came from the Catania metropolitan area and surrounding regions in Eastern Sicily. Participants were admitted to the Child Neurology and Psychiatry Unit, Department of Clinical and Experimental Medicine, University of Catania, Italy, during a 24-month period (1 October 2022–30 September 2024). All 102 individuals diagnosed with ASD met the DSM-5-TR diagnostic criteria [1], based on clinically accepted, standardized diagnostic instruments that include the Autism Diagnostic Observation Schedule (ADOS) and Autism Diagnostic Interview-Revised (ADI-R). The ADOS is a semi-structured, standardized tool designed to evaluate social affect (SA)—which includes language, communication and reciprocal social interaction—and restricted and repetitive behavior (RRB) in individuals suspected of ASD [19]. The ADI-R is a structured interview conducted by a trained examiner with a parent or caregiver, aimed at providing a detailed account of both developmental history and current functioning in domains associated with Autism Spectrum Disorder [20]. For individuals with ASD, exclusion criteria included the diagnosis of a monogenic disorder (such as Fragile X syndrome or Tuberous Sclerosis), abnormal findings on chromosomal microarray analysis, a documented history of mitochondrial disease or the presence of known medical conditions, such as autoimmune disorders and inflammatory gastrointestinal diseases, including celiac disease or Inflammatory Bowel Disease (IBD). Exclusion criteria also included use of ketogenic diets, restrictive diets and ARFID (Avoidant/Restrictive Food Intake Disorder). All participants in the study followed a Mediterranean diet and had a Body Mass Index (BMI) within the normal range for age and sex. None of the subjects were undergoing pharmacological treatment or engaged in any extraordinary physical activity during the study period. TD children were recruited among those performing routine blood analyses for screening iron-deficiency anemia, which was excluded in all included TD participants. All participants underwent a comprehensive clinical evaluation, including parental interview aimed at checking for present or past medical conditions, pharmacological therapies and laboratory investigations. Cognitive levels, emotional–behavioral disturbances and measures of the severity of ASD symptoms were assessed using a structured battery of neuropsychological assessments tailored to the participant’s age. In particular, either the Griffiths Scales of Child Development 3rd Edition (Griffiths III) [21,22] or the Wechsler Intelligence Scale for Children-4th Edition (WISC-IV) [23] was used to assess cognitive level. The ADOS Calibrated Severity Score (CSS) was used to obtain an estimate of the clinical severity of ASD [24], allowing comparison among different age groups. CSS TS (total score), SA (social affect) and RRB (repetitive and restricted behavior) were measured. Informed consent was obtained in writing from the parents of all participants prior to their inclusion in the study. This research represents a component of a broader project focused on identifying markers, predictors and developmental pathways of ASD. The comprehensive study received approval from the local ethics committee of the University Hospital Policlinico of Catania.

### 2.2. Sample Collections and ESI-MS/MS Analysis

To prevent any systematic bias related to sample collection timing, dried blood spots (DBSs) were obtained from both ASD and TD individuals using Whatman^TM^ 903 Proteinsaver Sample Collection Cards. Sampling was performed in the morning between 8:00 and 8:30 a.m. following an overnight fast. After drying, the DBSs were stored at 4 °C in a single refrigerator with controlled humidity and analyzed within two weeks of collection. For each participant, a 3.2 mm diameter blood spot was used for the analyses. Using electrospray ionization tandem mass spectrometry (ESI-MS/MS), the blood concentrations (μmol/L) of the following 31 ACs (satured, unsatured, hydroxylated and dicarboxylated) were analyzed simultaneously: free carnitine (C0); acetylcarnitine (C2:0); propionylcarnitine (C3:0); malonylcarnitine/hydroxybutyrylcarnitine (C3DC\C4OH); butyrylcarnitine (C4:0); methylmalonylcarnitine/3-hydroxyisovalerylcarnitine (C4DC\C5OH); valerylcarnitine (C5:0); glutarylcarnitine/3-hydroxycaproylcarnitine (C5DC\C6OH); tiglylcarnitine (C5:1); caproylcarnitine (C6:0); adipylcarnitine (C6DC); octanoylcarnitine (C8:0); octenoylcarnitine (C8:1); decanoylcarnitine (C10:0); decenoylcarnitine (C10:1); decadienoylcarnitine (C10:2); lauroylcarnitine (C12:0); dodecenoylcarnitine (C12:1); myristoylcarnitine (C14:0); tetradecenoylcarnitine (C14:1); tetradecadienoylcarnitine (C14:2); 3-hydroxy-tetradecanoylcarnitine (C14OH); palmitoylcarnitine (C16:0), hexadecenoylcarnitine (C16:1); 3-hydroxy-hexadecanoylcarnitine (C16OH); 3-hydroxy-hexadecenoylcarnitine (C16:1OH); stearoylcarnitine (C18:0); octadecenoylcarnitine (C18:1); octadecadienylcarnitine (C18:2); 3-hydroxysteroylcarnitine (C18OH); 3-hydroxyoleylcarnitine (C18:1OH). Each AC measurement represents a single reading per participant (biological replicate only).

### 2.3. Statistical Analysis

Blood levels of 31 targeted ACs (μmol/L) collected from 219 subjects, including 102 ASD patients and 117 TD healthy controls (HCs), were examined. The percentile distribution of blood AC levels in HCs was computed to define the normal reference range (RR) and identify the values corresponding to the 10th and 90th percentiles in the normal control sample. Because the AC panel contains multiple measurements, we identified the AC panel having three or more abnormal ACs (AC profile) compared to reference range values in two consecutive blood samples as previously reported [14] Then, we classified ASD patients into 3 groups: (1) group A (≥3 analytes <10th pc), (2) group B (all analytes in the RR) and (3) group C (≥3 analytes >90th pc). Categorical data were reported as counts and percentages, while continuous data were summarized using means and standard deviations. The distribution of continuous variables was evaluated using the Shapiro–Wilk test to assess normality. Since the quantitative variables did not follow a normal distribution, the Kruskal–Wallis test was employed to compare the three groups. In cases where the Kruskal–Wallis test indicated significant differences, post hoc pairwise comparisons were performed with Bonferroni correction applied to adjust for multiple testing. Subsequently, we classified ASD patients based on (1) the presence/absence of intellectual disability (ID)/developmental delay (DD) and (2) the severity of ASD symptoms measured by the CSS. Pearson’s chi-square tests were utilized to compare categorical variables and to examine the proportion of participants exhibiting abnormal AC levels across ASD clinical groups. We used Yates’ continuity correction to adjust the chi-squared test statistic to improve the accuracy of the analysis. Since all continuous variables deviated from normality, the Mann–Whitney U test was employed to assess differences in blood AC levels according to the following clinical features: (1) the presence/absence of ID (ASD-wID vs. ASD-w/oID) and (2) ASD severity (ADOS CSS ≤ 7 or CSS ≥ 8). The data analysis was conducted using SPSS software version 27 (SPSS, Inc., Chicago, IL, USA; IBM, Somers, NY, USA). Statistical significance was set at a *p*-value less than 0.05.

## 3. Results

### 3.1. Blood AC Levels in ASD and TD Participants

The percentile distribution of blood AC levels in TD was computed to define the normal reference range (RR) and identify the values corresponding to the 10th and 90th percentiles in the normal control sample. Based on the prevalent blood AC profile, three ASD subgroups were detected. Specifically, the first consisted of those who had blood ACs levels less than the 10th percentile of the RR (males: 20, females: 6; age 7.0 ± 4.2 years); the second group, the most numerous one, consisted of those who had ACs levels in the normal RR (males: 35, females: 7; age 6.2 ± 3.3 years); the third group included ASD patients with ACs levels greater than the 90th percentile in the considered RR (males: 29, females: 5; age 5.5 ± 3.0 years). A total of four patients were identified, exhibiting both low AC levels falling below the 10th percentile and elevated AC levels above the 90th percentile. In order to circumvent the violation of the assumption of independent group comparisons, based on the prevalent AC profile (<10th percentile), the four patients were exclusively assigned to group A. For simplicity, we will refer to three study groups based on the AC profile: group A (<10th pc; *n*: 26, 25.5%), group B (10th–90th pc; *n*: 42, 41.2%) and group C (>90th pc; *n*: 34, 33.3%).

### 3.2. Clinical Characteristics

Notably, the three ASD study groups (A–C) had homogeneous demographic features, ensuring a balanced comparison for subsequent analyses (Table 1). Statistical analysis revealed no significant differences relating to age (*p* = 0.474) and gender distribution (*p* = 0.684). Moreover, the rate of non-verbal ASD (*p* = 0.147), microcephaly (*p* = 0.828) and macrocephaly (*p* = 0.277) among the three study groups was not significantly different. In order to highlight possible clinical correlates of blood AC profiles, we considered differences in the rate or extent of several clinical comorbidities among the three study groups identified in the blood AC profiles. No statistically significant differences were detected regarding the proportion of participants with regressive ASD (*p* = 0.126), intellectual disability (*p* = 0.385), EEG anomalies (*p* = 0.751) and epilepsy (*p* = 0.486) (Table 1). Out of the 102 ASD patients, intellectual disability (ID) was diagnosed in 69 (67.6%) (ASD-wID), while 33 patients (32.4%) had a normal cognitive level (ASD-w/oID). Comparison of blood AC levels between w-ID and w/o-ID groups showed no significant differences. Thirty-six patients (35.3%) had more severe autism symptoms (CSS 8–10) and sixty-six (64.7%) had milder symptoms (CSS 4–7). No significant differences in blood AC levels were observed between ASD patients with different ASD severity measured by total CSS (≤7 and ≥8). We then evaluated possible differences in the severity of ASD symptoms measured by the ADOS-2 scores and the ADOS CSS, i.e., CSS SA, CSS RRB and CSS total among the three study groups (Table S1). The Kruskal–Wallis test indicated a significant difference in the dependent variable CSS RRB among the different groups, χ^2^(2) = 6.26, *p* = 0.044, with a mean rank score of 61.33 for Group 1, 43.7 for Group 2 and 53.62 for Group 3. The post hoc Dunn’s test using a Bonferroni-corrected alpha of 0.017 indicated that the severity score related to the repetitive and restricted behavior domain (CSS RRB) was higher in ASD group A compared to ASD group B (*p* = 0.013).

### 3.3. Clinical Correlates of Individual Blood AC in Patients with ASD

To further evaluate the possible clinical correlates of blood AC profiles in patients with ASD, the analysis centered on the proportion (%) of subjects with abnormal values for each individual AC in each group (Table 2). Considering ASD group A (<10th percentile), the most frequently abnormal ACs (<10th pc) were C3DC\C4OH (*n* = 9, 34.6%), C10:2 (*n* = 18, 69.2%), C14OH (*n* = 11, 42.3%), C16OH (*n* = 9, 34.6%) and C18OH (*n* = 14, 53.8%). Considering ASD group C (>90th percentile), the most frequently abnormal ACs were C0 (*n* = 17, 50%), C2 (*n* = 14, 41.2%), C3 (*n* = 16, 47%), C4 (*n* = 13, 38.2%), C4DC\C5OH (*n* = 15, 44.1%), C8 (*n* = 9, 26.5%), C10 (*n* = 18, 52.9%), C14:2 (*n* = 11, 32.35%), C16 (*n* = 10, 29.4%), C16:1 (*n* = 13, 38.2%), C18:1 (*n* = 19, 55.9%) and C18:2 (*n* = 10, 29.4%).

Out of the thirty-one analyzed ACs in DBSs, fifteen (48.4%) were significantly different when comparing ASD group A to ASD group C (Table 2). The abnormal ACs in ASD patients included five short-chain (2–5 carbon length) ACs (C0: *p*-value 0.0001; C3: *p*-value 0.003; C4: *p*-value 0.0004; C4DC\C5OH: *p*-value 0.002; C5:1: *p*-value 0.007), three medium-chain (6–12 carbon length) ACs (C8: *p*-value 0.004; C10: *p*-value 0.0001; C10:2: *p*-value < 0.0001), and seven long-chain (13–18 carbon length) ACs (C14:2: *p*-value 0.02; C14OH: *p*-value < 0.0001; C16:1: *p*-value 0.0004; C16OH: *p*-value 0.013; C18:1: *p*-value < 0.0001; C18:2: *p*-value 0.002; C18OH: *p*-value < 0.0001). Statistical significance was confirmed using Yates’ correction for continuity (Table 2).

**Figure 2:**
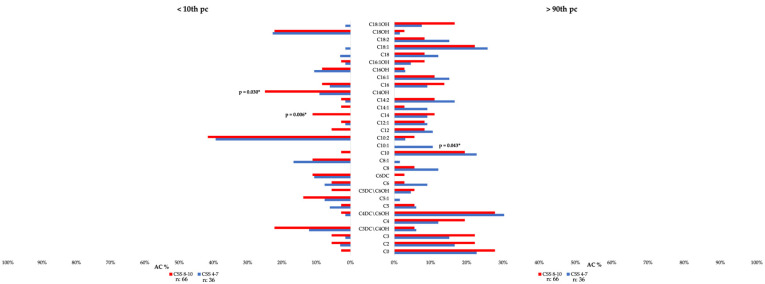
Distribution of patients (%) with AC values below (<10th pc) and above (>90th pc) the reference range according to the severity of ASD measured by ADOS-CSS. CSS: ADOS Calibrated Severity Score; *: statistically significant difference (*p* < 0.05).

Then we searched whether the severity of ASD (CSS) was different in patients according to individual blood AC levels (Figure 2, Table S2).

We found that patients with more severe ASD (CSS 8–10) more frequently presented decreased values (below the 10th pc) of C14 (*p* = 0.006) and C14:OH (*p* = 0.03). Following the implementation of Yates’ correction, only C14 values retained statistical significance (Table S3). In contrast, C10:1 levels were found to be more elevated (above the 90th pc) in patients with lower clinical severity (lower CSS) compared to those with more severe ASD (CSS 8–10), although the difference was not significant after multiple comparison correction (*p* = 0.052) (Table S4).

## 4. Discussion

ASD is a multifactorial condition, stemming from the combined effects of genetic risk and environmental factors [25]. Gaining deeper insight into the molecular mechanisms underlying ASD clinical variability is crucial for advancing screening methods, refining diagnostic tools and developing personalized therapeutic strategies [26]. In the present study, we systematically analyzed blood AC levels in ASD children with reference to the normal percentile distribution in healthy participants with the same age and sex distribution. A subset of ASD participants had an increase (>90th pc) in the number (*N* ≥ 3) of various ACs, such as short-chain (C0, C3, C4, C4DC/C5OH and C5:1), medium-chain (C8 and C10) and long-chain ACs (C14:2, C16:1, C18:1 and C18:2), consistent with previous controlled studies [7,12,14,16]. Notably, a subset of study children with ASD (25.5%) had abnormal ACs levels (*N* ≥ 3) below the normal range (i.e., C10:2, C14OH, C16OH and C18OH), with medium-chain C10:2 decreased in almost 70% of these patients. In this regard, it was found that Chinese pre-school children with ASD had significantly lower levels of short (C5DC), medium (C8:1) and very long (C24:1, C26:1) ACs compared to healthy age-matched controls [27]. Notably, the same research identified a significant positive association between octenoyl carnitine (C8:1) concentrations and the total and performance IQ scores in ASD children. In the present study, out of the thirty-one analyzed blood ACs, fifteen (48.4%) were abnormal in a proportion of studied patients. Abnormal ACs in children with ASD more frequently included an increase in short-chain ACs and long-chain ACs, and frequently increased (C8, C10) or decreased (C10:2) medium-chain ACs. This is consistent with a systematic review and metanalysis of mitochondrial biomarkers in ASD comparing ASD children with and without AC elevations demonstrating notable differences in a number of short-chain ACs (such as C5:0, C4DC\C5OH, C5DC and C6:0), various medium-chain ACs (including C8:0, C10:0, C10:1, C12:0 and C12:1) and several long-chain ACs (such as C14:0, C14:1, C14:2, C14OH, C14:1OH, C16:1, C16OH and C18:0) [7]. Interestingly, a recent study conducted by Ahrens et al. [28] revealed that in the stool of infants who were developing ASD, C16:1 levels were higher compared to a group of neurotypical infants, supporting the increase in C16:1 in the blood of older children with ASD ([12,14], present study). Likewise, ACs were identified as significant in four out of the thirty clusters of metabolic biomarkers discovered within a diagnostic algorithm for ASD [29]. Finally, among 73 studied adults with ASD, a distinct AC profile was observed, characterized by an increase in short- and medium-chain ACs, while long-chain C14:2 AC, typically higher in children, was found to be lower [30]. This might indicate a metabolic status shift in ASD that occurs with advancing age. Based on the previous and present results, we suggest that both increased and decreased individual AC values may occur in patients with ASD, indicating potential mitochondrial dysfunction. Alterations in blood AC levels have been linked to neurodevelopmental regression in a study conducted on children with ASD [13] and have also been associated with key ASD symptoms, empathy levels and depression in adults diagnosed with ASD [30]. Our analysis revealed no statistically significant differences among the three study groups regarding the recurrence of regression, ID, EEG abnormalities and epilepsy, according to AC profiles. This suggests that in the study patients, the blood AC profile does not directly correlate with regressive symptoms nor with some ASD comorbid conditions. However, considering ASD clinical severity, it was evident that subjects with multiple AC values out of the RR, namely those belonging to group A (<10th pc) compared to group B (AC levels in the normal range), exhibit more severe autistic features related to the RRB domain score on the ADOS-2. This domain encompasses a wide range of behaviors and interests that are characterized by repetition, rigidity and a narrowed focus, such as stereotyped or repetitive motor movements, adherence to sameness, ritualistic behaviors, restricted range of interests and hyper- or hyporeactivity to sensory input [31]. We also observed that 69.2% of patients in group A showed reduced levels of C10:2, compared to only 11.8% in group C. Decanoic acid (C10) supplementation in vitro improves ETC and mitochondrial proliferation in neuronal cell lines, supporting its role in ameliorating brain energy metabolism. Moreover, C10 activates the peroxisome proliferator-activated receptor gamma, promoting the formation of new mitochondria and boosting antioxidant defenses [32]. Considering this, recognizing metabolic subgroups based on AC profiling may allow for the design of targeted dietary interventions aimed at correcting underlying nutrient deficiencies. Dietary intervention with a unique ratio of decanoic acid to octanoic acid was effective in reducing epileptic seizures (50%) in patients with drug-resistant epilepsy, and this correlated significantly with blood levels of medium-chain fatty acids (C10 and C8) [33]. Evaluating the blood AC profile in ASD patients both at baseline and following proper dietary interventions would be crucial in elucidating the potential beneficial effects of improving energy metabolism on the clinical correlates.

Furthermore, the complex relationship between genetics and environmental factors influencing blood AC levels in patients with ASD is unclear as yet. Previous research suggested that enteric short-chain fatty acids, such as propionic acids, might act as environmental triggers of mitochondrial dysfunction in genetically predisposed subjects with ASD. Actually, such enteric short-chain fatty acids are produced by microbiota species frequently associated with ASD (i.e., *Clostridium bacteria* and *Desulfovibrio species)*. It was hypothesized that excessive enteric propionic acid might be converted to propionil-CoA and methylmalonil-CoA, thus entering into the trycarboxilic acid cycle (TCA). This might ultimately inhibit the proximal half of the TCA in terms of energy efficiency and cause the accumulation of acetyl-CoA, which represents the final product of the mFAO cycle [14]. Although clinical studies evaluating the use of prebiotics or probiotics in ASD for alleviating gastrointestinal and behavioral symptoms yielded controversial results, it was suggested that future, more mechanistic trials might integrate metabolomic data and a more standardized intervention regimen [34]. Moreover, environmental factors might modulate the mFAO activity through the peroxisome proliferator-activated receptors (PPARs). These nuclear receptors play a crucial role in regulating mitochondrial function and inflammatory responses. The activation of PPARs leads to the upregulation of numerous FAO enzymes, resulting in increased mFAO flux and improved energy homeostasis, pathways often disrupted in ASD [7,16,35]. In this context, RSV, a natural polyphenolic compound, has emerged as a promising therapeutic candidate. Known for its antioxidant, anti-inflammatory, metabolic and neuroprotective properties, RSV also functions as a natural agonist of PPARs. Several preclinical and pilot clinical studies have highlighted its therapeutic potential across a range of pathologies, including ASD [16,34]. Importantly, RSV has been shown to stimulate mFAO in control human fibroblasts and restore normal mFAO rates in fibroblasts from individuals with mild inborn mFAO deficiencies. A recent study revealed significant differences in basal mFAO activity in fibroblasts from ASD individuals, depending on the presence or absence of elevated blood ACs. Moreover, RSV treatment led to a significant increase in mFAO activity, with the most pronounced effects observed in the most severely affected patients. This suggests that RSV may exert its beneficial effects through the correction of underlying mitochondrial dysfunctions [35]. These findings support a possible therapeutic effect of RSV in ASD patients, particularly those with metabolic abnormalities. Given this, it would be of considerable interest to conduct further research exploring targeted interventions based on individual metabolic profiles. Understanding how distinct metabolic phenotypes influence treatment response could pave the way for more personalized and effective therapeutic strategies in ASD.

While our study focuses primarily on mFAO as reflected by plasma AC profiles, it is important to consider the upstream metabolic contributions of peroxisomes to fatty acid metabolism. Peroxisomes play a fundamental role in the initial catabolism of very-long-chain fatty acids (VLCFAs) with a chain length >C22 (26:0, C26:1, C24:0/C22:0 and C26:0/C22:0), branched-chain fatty acids and certain dicarboxylic acids, which are subsequently shuttled to mitochondria for complete oxidation [36,37]. This peroxisome–mitochondria metabolic crosstalk is essential for maintaining lipid homeostasis and energetic balance [37,38]. Deficits in peroxisomal function may impair the processing and availability of substrates for mFAO, leading to secondary mitochondrial dysfunction and altered AC patterns [37,38]. Such dysfunctions have been previously associated with ASD, suggesting that metabolic abnormalities observed in ASD may originate from or be compounded by upstream peroxisomal impairment [39]. In light of this, the altered AC profiles observed in our cohort may reflect not only mitochondrial dysregulation but also upstream disruptions in peroxisomal fatty acid processing. Future studies integrating peroxisomal biomarkers, VLCFAs and enzyme activity measurements could help clarify the respective contributions of these organelles to the metabolic phenotype observed in ASD.

It is important to recognize the limitations when interpreting the findings of this study. One notable constraint is the relatively small sample size of participants with ASD, which may affect the generalizability of the results to the broader ASD population. Additionally, the study design provides only a single snapshot of AC profiles and clinical characteristics, limiting the ability to observe changes or developments over time. This temporal limitation restricts our understanding of the dynamic nature of these metabolic markers and how they may fluctuate in relation to the progression or variation in ASD symptoms. To gain a deeper and more thorough understanding of these markers and their possible links to ASD symptoms, longitudinal research would be required [40]. Moreover, the relatively small sample size and stringent inclusion criteria prevented us from considering additional clinical variables such as gastrointestinal symptoms, feeding patterns, sleep disturbances or physical activity, all of which may influence or be influenced by metabolic profiles. Further analysis in larger samples might take advantage of stratified or adjusted models considering the sex, BMI and pubertal stage of the participants to improve data interpretability. The definition of group A and group C as ≥3 ACs below the 10th percentile and ≥3 ACs above the 90th percentile, respectively, could not ensure mutually exclusive categories in all instances. As a matter of fact, we identified four patients with various AC levels falling below the 10th percentile and a minor number of AC above the 90th percentile. In order to circumvent the violation of the assumption of independent group comparisons, based on the prevalent AC profile (<10th percentile), we exclusively assigned the four patients to group A.

## 5. Conclusions

The results of this study reveal that abnormal blood AC profiles may occur in ASD, involving increased or decreased levels of multiple ACs in a proportion of patients and may be associated with the severity of autistic features. Thus, examining metabolic profiles may provide helpful insights to study the clinical variability of ASD. Future studies should expand our observations to understand the impact of additional clinical variables on AC metabolism in patients with ASD.

## Figures and Tables

**Table 1:** Demographic and clinical characteristics of ASD patient groups (A–C) based on blood AC profile (≥3 abnormal AC levels).

*ASD Group* *N (%)*	*A* *(n: 26)*	*B* *(n: 42)*	*C* *(n: 34)*	*p-Value*
*Age at diagnosis*	3.4 ± 1.0	3.4 ± 0.9	3.3 ± 0.9	0.812
*Age at sample collection*	7 ± 4.2	6.2 ± 3.3	5.5 ± 3.0	0.474
*Sex (Male)*	20 (76.9%)	35 (83.3%)	29 (85.3%)	0.684
*Non-verbal ASD*	2 (7.7%)	3 (7.1%)	7 (20.6%)	0.147
*Microcephaly*	2 (7.7%)	5 (11.9%)	3 (8.8%)	0.828
*Macrocephaly*	2 (7.7%)	8 (19.05%)	3 (8.8%)	0.277
*Regression*	10 (38.5%)	7 (16.7%)	8 (23.5%)	0.126
*IQ/DQ*	62.3 ± 13.8	60.5 ± 18.4	60.8 ± 16.7	0.278
*ID*	15 (57.7%)	31 (73.8%)	23 (67.6%)	0.385
*EEG anomalies*	3 (11.5%)	3 (7.1%)	4 (11.8%)	0.751
*Epilepsy*	0 (0%)	1 (2.4%)	0 (0%)	0.486

ASD Group A: AC levels <10th pc; ASD Group B: AC levels between 10th and 90th pc; ASD Group C: AC levels >90th pc; IQ: Intelligence Quotient; DQ: Developmental Quotient; ID: Intellectual Disability; EEG: Electroencephalogram.

**Table 2:** Statistically significant differences in blood AC in ASD group A compared to ASD group C.

*N* (%)	ASD Group A (*n* = 26)	ASD Group C (*n* = 34)	*p*-Value	*p* (Yates’ Correction)
C0	1 (3.8%)	17 (50%)	0.0001 *	0.0003 *
C2	4 (15.4%)	14 (41.2%)	0.0307	0.0606
C3	3 (11.5%)	16 (47.0%)	0.003 *	0.008 *
C3DC\C4OH	9 (34.6%)	5 (14.7%)	0.071	0.134
C4	0 (0%)	13 (38.2%)	**0.0004** *	0.001 *
C4DC\C5OH	2 (7.7%)	15 (44.1%)	**0.0019** *	0.005 *
C5	4 (15.4%)	3 (8.8%)	0.433	0.705
C5:1	7 (26.9%)	1 (2.9%)	**0.007** *	0.02 *
C5DC\C6OH	6 (23.1%)	3 (8.8%)	0.125	0.243
C6	7 (26.9%)	7 (20.5%)	0.565	0.79
C6DC	3 (11.5%)	1 (2.9%)	0.186	.423
C8	0 (0%)	9 (26.5%)	**0.004** *	0.013 *
C8:1	4 (15.4%)	1 (2.9%)	0.084	0.2087
C10	1 (3.8%)	18 (52.9%)	**0.00005** *	0.0002 *
C10:1	0 (0%)	6 (17.6%)	**0.024** *	0.068
C10:2	18 (69.2%)	4 (11.8%)	<0.0001 *	**<0.0001**
C12	2 (7.7%)	9 (26.5%)	0.062	0.127
C12:1	2 (7.7%)	6 (17.6%)	0.261	0.459
C14	2 (7.7%)	5 (14.7%)	0.402	0.665
C14:1	1 (3.8%)	6 (17.6%)	0.0989	0.213
C14:2	2 (7.7%)	11 (32.35%)	0.0216 *	0.0475 *
C14OH	11 (42.3%)	0 (0%)	<0.0001 *	<0.0001 *
C16	6 (23.1%)	10 (29.4%)	0.583	0.799
C16:1	0 (0%)	13 (38.2%)	0.0004 *	0.001 *
C16OH	9 (34.6%)	3 (8.8%)	0.013 *	0.03 *
C16:1OH	1 (3.8%)	3 (8.8%)	0.444	0.808
C18	1 (3.8%)	8 (23.5%)	**0.034** *	0.0799
C18:1	0 (0%)	19 (55.9%)	<0.0001 *	<0.0001 *
C18:2	0 (0%)	10 (29.4%)	0.002 *	0.007 *
C18OH	14 (53.8%)	2 (5.9%)	<0.0001 *	<0.0001 *
C18:1OH	1 (3.8%)	8 (23.5%)	**0.034** *	0.0799

ASD Group A: AC levels <10th pc; ASD Group C: AC levels >90th pc; *: statistically significant difference (*p* < 0.05)

## Data Availability

The original contributions presented in this study are included in the article/Supplementary Materials. Further inquiries can be directed to the corresponding author.

## Supplementary Materials

The following supporting information can be downloaded at: https://www.mdpi.com/article/10.3390/children12070848/s1, Table S1: Autism severity scores in ASD study groups. Table S2: Patients (n) with AC values below (<10th pc) and above (>90th pc) the reference range according to the severity of ASD measured by ADOS-CSS. Table S3: Distribution of patients with below normal AC values (<10th pc) according to the severity of ASD. Table S4. Distribution of patients with upper normal AC values (>90th pc) according to the severity of ASD.
