# Supplementary material for: Clinical and Behavioral Correlates of Blood Acylcarnitine Profiles in Children with Autism Spectrum Disorder: A Cross-Sectional Analysis
Source: Children (Basel). 2025 Jun 27;12(7):848. doi: 10.3390/children12070848 (PMC12293323; doi:10.3390/children12070848)
Supplement: Supplementary file 1 [file children-12-00848-s001.zip › children-3652953-supplementary.pdf]

SUPPLEMENTARY MATERIAL

Table S1: Autism severity scores in ASD study groups

	ASD group A (n: 26)	ASD group B (n: 42)	ASD group C (n: 34)	*p*-value
ADOS-2 SA	14.15 ± 5.1	13.6 ± 5.6	13.8 ± 3.9	0.706
ADOS-2 RRB	3.4 ± 1.6	2.6 ± 1.8	3 ± 1.3	0.144
ADOS-2 TS	17.6 ± 5.5	16.2 ± 6.1	16.8 ± 4.7	0.571
ADOS-CSS SA	7.5 ± 2.0	7.4 ± 2.5	7.6 ± 2.2	0.907
ADOS-CSS RRB	6.8 ± 1.4	5.6 ± 2.1	6.4 ± 1.4	**0.044***
ADOS-CSS TS	6.96 ± 1.8	6.7 ± 2.4	7.1 ± 1.9	0.864

N patients=102.

ASD Group A: AC levels <10th pc; ASD Group B: AC levels between 10th-90th pc; ASD Group C: AC levels >90th pc. ADOS-2: *Autism Diagnostic Observation Schedule – Second Edition*; ADOS-CSS: *ADOS Calibrated Severity Score*; SA, social affect; RRB, repetitive and restricted behaviors; TS, total score; *= statistically significant difference ($p < 0.05$). p-value refers to Kruskal-Wallis tests in case of quantitative variables.

Table S2: Patients (n) with AC values below (< 10th pc) and above (> 90th pc) the reference range according with the severity of ASD measured by ADOS-CSS.

	**< 10th pc**		**> 90th pc**	
	CSS 4-7 (n)	CSS 8-10 (n)	CSS 4-7 (n)	CSS 8-10 (n)
C0	0	1	15	10
C2	2	2	11	8
C3	1	2	10	8
C3DC\C4OH	8	8	4	2
C4	0	0	8	7
C4DC\C6OH	1	1	20	10
C5	4	1	4	2
C5:1	5	5	1	0
C5DC\C6OH	4	2	3	2
C6	5	2	6	1
C6DC	7	4	0	1
C8	0	0	8	2
C8:1	11	4	1	0
C10	0	1	15	7
C10:1	0	0	7	0
C10:2	26	15	2	2
C12	0	2	7	3
C12:1	1	1	6	3
**C14**	0	4	6	4
C14:1	0	1	6	1
C14:2	1	1	11	4
**C14OH**	6	9	0	0
C16	4	3	6	5
C16:1	0	0	10	4
C16OH	7	3	2	1
C16:1OH	1	1	3	3
C18	2	0	8	3
C18:1	1	0	17	8
C18:2	0	0	10	3
C18OH	15	8	1	1
C18:1OH	1	0	5	6

ADOS CSS: *ADOS Calibrated Severity Score*

Table S3: Distribution of patients with below normal AC values (< 10th pc) according with the severity of ASD. Different shades of grey subdivide acylcarnitines according to their length, into short-, medium-, and long-chain acylcarnitines. CSS: *ADOS Calibrated Severity Score.*

	CSS 4-7 (n=66)	CSS 8-10 (n=36)	*p*-value	*p (Yates Correction)*
C0	0 (0%)	1 (2,77%)	0.174	0.757
C2	2 (3,03%)	2 (5,55%)	0.530	0.924
C3	1 (1,51%)	2 (5,55%)	0.248	0.588
C3DC\C4OH	8 (12,12%)	8 (22,22%)	0.180	0.291
C4	0 (0%)	0 (0%)	1	0.916
C4DC\C6OH	1 (1,51%)	1 (2,77%)	0.660	0.758
C5	4 (6,06%)	1 (2,77%)	0.463	0.799
C5:1	5 (7,57%)	5 (13,8%)	0.305	0.499
C5DC\C6OH	4 (6,06%)	2 (5,55%)	0.917	0.737
C6	5 (7,57%)	2 (5,55%)	0.7	0.975
C6DC	7 (10,60%)	4 (11,11%)	0.937	0.799
C8	0 (0%)	0 (0%)	1	0.916
C8:1	11 (16,66%)	4 (11,11%)	0.449	0.642
C10	0 (0%)	1 (2,77%)	0.174	0.757
C10:1	0 (0%)	0 (0%)	1	0.916
C10:2	26 (39,39%)	15 (41,66%)	0.823	1
C12	0 (0%)	2 (5,55%)	0.053	0.235
C12:1	1 (1,51%)	1 (2,77%)	0.660	0.758
**C14**	0 (0%)	4 (11,11%)	**0.006***	**0.0258***
C14:1	0 (0%)	1 (2,77%)	0.174	0.757
C14:2	1 (1,51%)	1 (2,77%)	0.660	0.758
C14OH	6 (9,09%)	9 (25%)	**0.030***	0.061
C16	4 (6,06%)	3 (8,33%)	0.664	0.975
C16:1	0 (0%)	0 (0%)	1	0.916
C16OH	7 (10,60%)	3 (8,33%)	0.712	1
C16:1OH	1 (1,51%)	1 (2,77%)	0.660	0.758
C18	2 (3,03%)	0 (0%)	0.291	0.758
C18:1	1 (1,51%)	0 (0%)	0.458	0.757
C18:2	0 (0%)	0 (0%)	1	0.916
C18OH	15 (22,72%)	8 (22,22%)	0.953	0.849
C18:1OH	1 (1,51%)	0 (0%)	0.458	0.757

Table S4. Distribution of patients with upper normal AC values (>90th pc) according with the severity of ASD. Different shades of grey subdivide acylcarnitines according to their length, into short-, medium-, and long-chain acylcarnitines. CSS: *ADOS Calibrated Severity Score.*

	CSS 4-7 (n=66)	CSS 8-10 (n=36)	*p*-value	*p (Yates Correction)*
C0	15 (22,72%)	10 (27,77%)	0.571	0.745
C2	11 (16,66%)	8 (22,22%)	0.491	0.672
C3	10 (15,15%)	8 (22,22%)	0.371	0.533
C3DC\C4OH	4 (6,06%)	2 (5,55%)	0.917	0.737
C4	8 (12,12%)	7 (19,44%)	0.318	0.48
C4DC\C6OH	20 (30,30%)	10 (27,77%)	0.789	0.964
C5	4 (6,06%)	2 (5,55%)	0.917	0.737
C5:1	1 (1,51%)	0 (0%)	0.458	0.757
C5DC\C6OH	3 (4,54%)	2 (5,55%)	0.821	0.799
C6	6 (9,09%)	1 (2,77%)	0.228	0.426
C6DC	0 (0%)	1 (2,77%)	0.174	0.757
C8	8 (12,12%)	2 (5,55%)	0.287	0.473
C8:1	1 (1,51%)	0 (0%)	0.458	0.757
C10	15 (22,72%)	7 (19,44%)	0.7	0.893
C10:1	7 (10,60%)	0 (0%)	**0.043***	**0.052***
C10:2	2 (3,03%)	2 (5,55%)	0.530	0.924
C12	7 (10,60%)	3 (8,33%)	0.712	1
C12:1	6 (9,09%)	3 (8,33%)	0.897	0.813
C14	6 (9,09%)	4 (11,11%)	0.743	1
C14:1	6 (9,09%)	1 (2,77%)	0.228	0.426
C14:2	11 (16,66%)	4 (11,11%)	0.449	0.642
C14OH	0 (0%)	0 (0%)	1	0.916
C16	6 (9,09%)	5 (13,8%)	0.455	0.68
C16:1	10 (15,15%)	4 (11,11%)	0.571	0.79
C16OH	2 (3,03%)	1 (2,77%)	0.942	0.588
C16:1OH	3 (4,54%)	3 (8,33%)	0.437	0.737
C18	8 (12,12%)	3 (8,33%)	0.556	0.799
C18:1	17 (25,75%)	8 (22,22%)	0.692	0.877
C18:2	10 (15,15%)	3 (8,33%)	0.324	0.499
C18OH	1 (1,51%)	1 (2,77%)	0.660	0.758
C18:1OH	5 (7,57%)	6 (16,66%)	0.157	0.28
